# Supplementary material for: Hog1 acts in a Mec1-independent manner to counteract oxidative stress following telomerase inactivation in Saccharomyces cerevisiae
Source: Commun Biol. 2024 Jun 22;7:761. doi: 10.1038/s42003-024-06464-3 (PMC11193714; doi:10.1038/s42003-024-06464-3)
Supplement: Supplementary file 2 — Supplementary Information [file 42003_2024_6464_MOESM2_ESM.pdf]

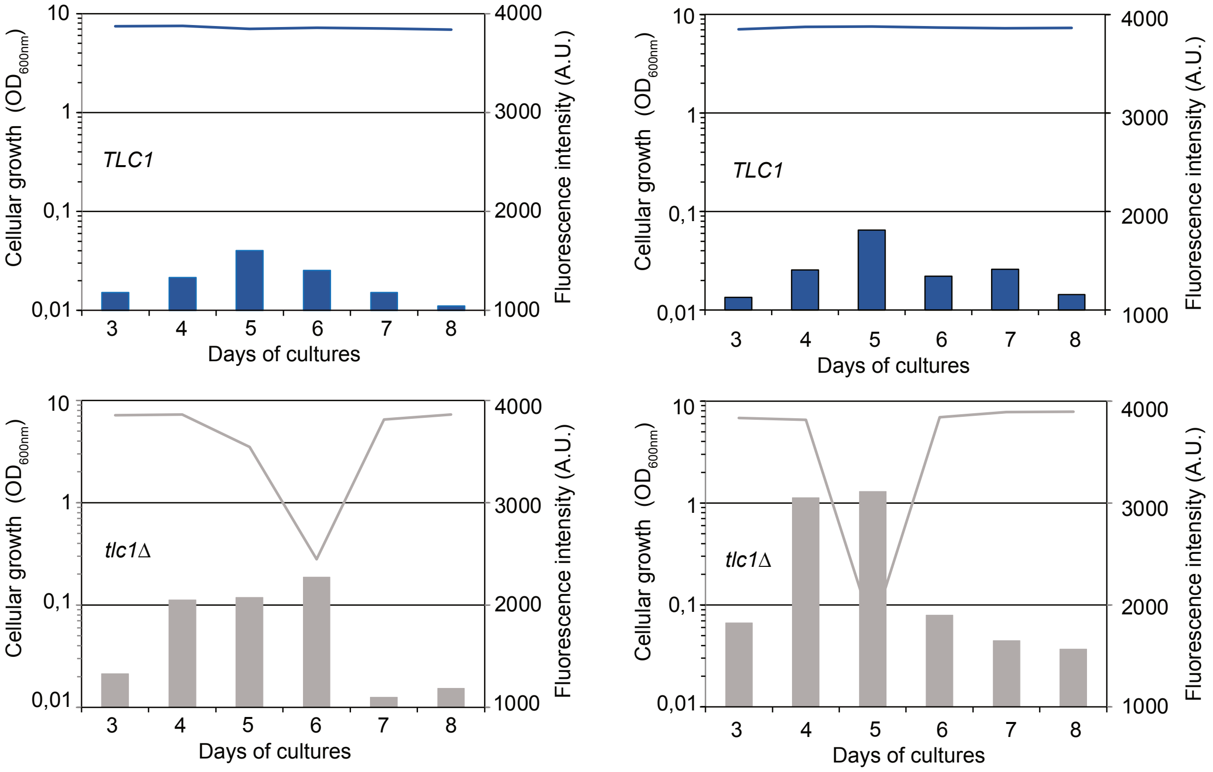

Supplementary Figure 1: ROS levels increase during replicative senescence in *tlc1*Δ strains. A diploid *TLC1/tlc1*Δ strain was dissected, and the four spore-derived colonies obtained after two days were pre-cultured . Each consecutive day, cultures were diluted, as described in Figure 1, and grown for 24h. Each graph represents the cellular growth and ROS levels measured for each of four haploids derived from the same tetrad. Cell density at OD_{600nm} (curve-left axis) and ROS levels (histogram-right axis) are plotted.

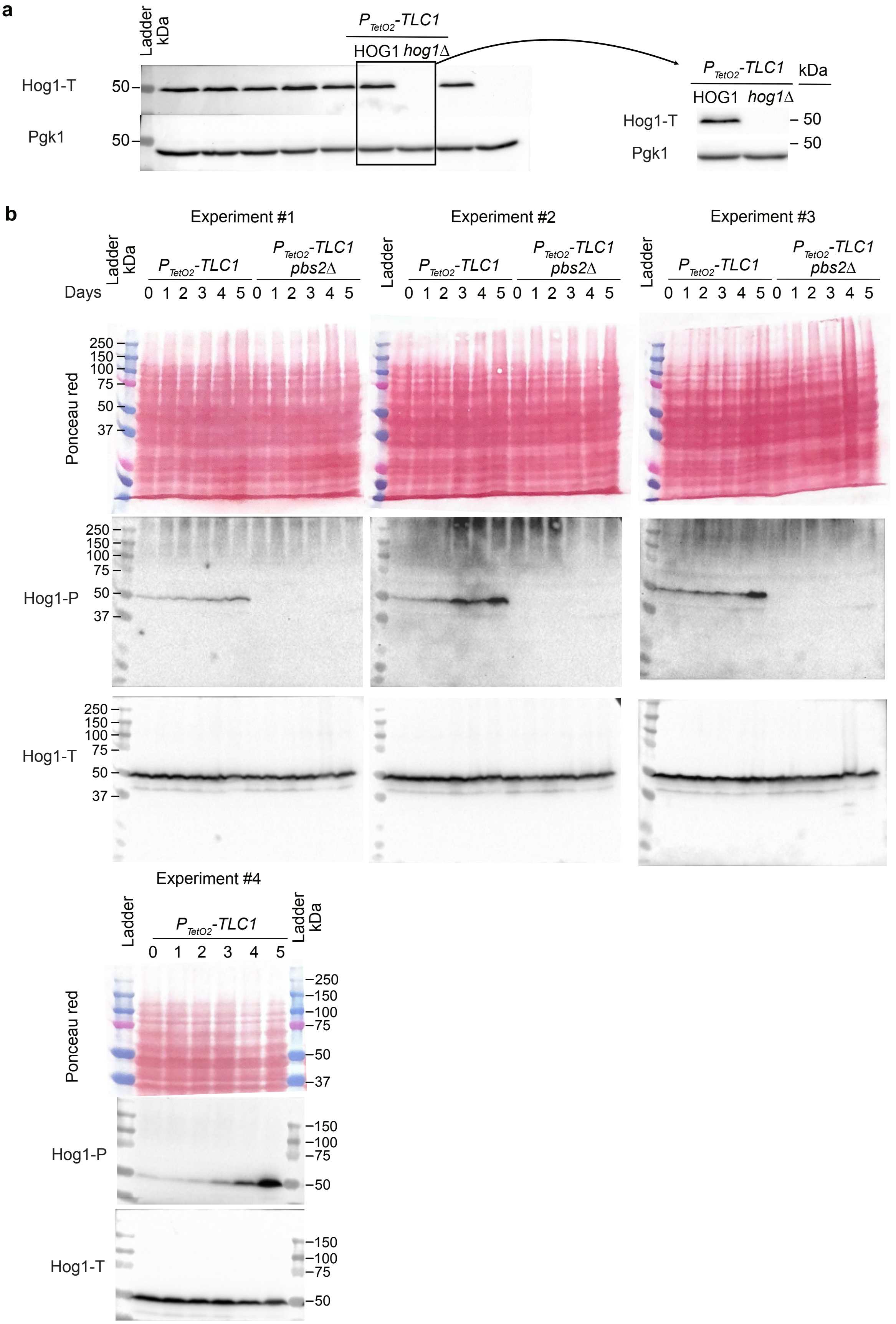

Supplementary Figure 2: Uncropped scans of Western blot. (a) Uncropped scans of Western blot relative to Figure 2c. Protein extracts analysed by Western blot using an antibody against the total Hog1 form (Hog1-T) or Pgk1. **(b) Uncropped scans of Western blot of 4 independent experiments relative to Figure 2d.** Protein extracts analysed by Western blot using an antibody against the phosphorylated forms of Hog1 human ortholog p38 (Hog1-P), or total Hog1 (Hog1-T).

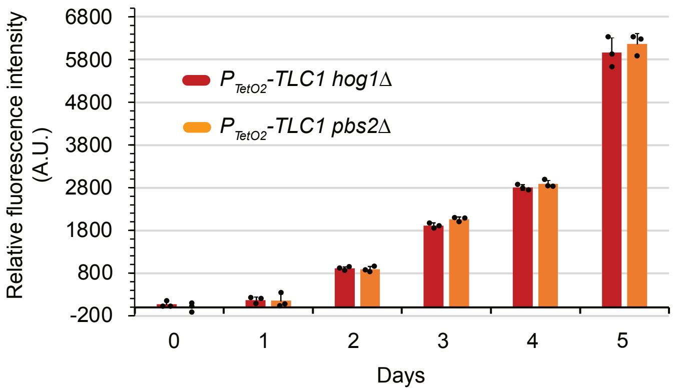

Supplementary Figure 3: ROS level during replicative senescence in absence of *HOG1* or *PBS2*. Cells with the genotypes indicated were treated as described in Figure 1. ROS levels are normalized to the P_{TetO2}-*TLC1* strain without doxycycline and plotted as mean ± SD of three independent experiments.

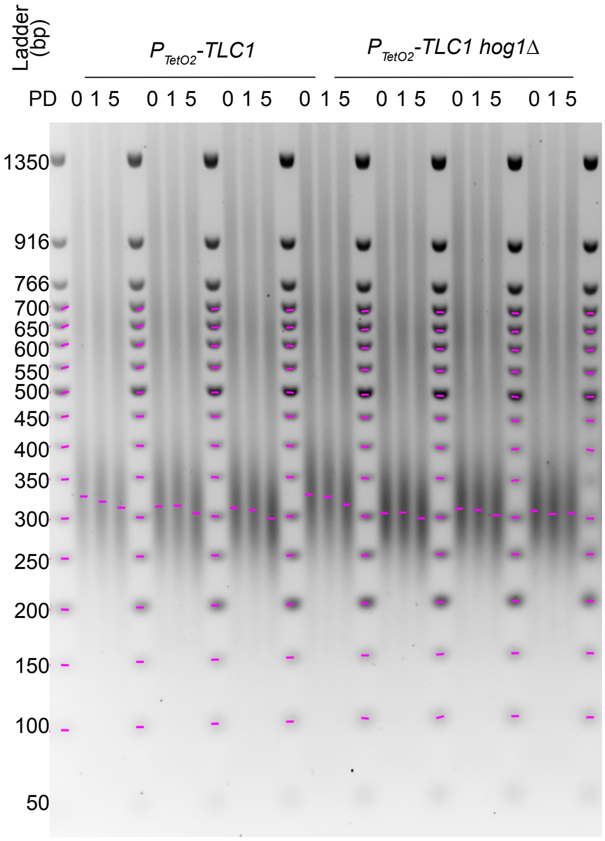

Supplementary Figure 4: *HOG1* deletion doesn't affect telomere shortening rate. Technical triplicates of the experiment described in Figure 3a. Population doublings (PD).

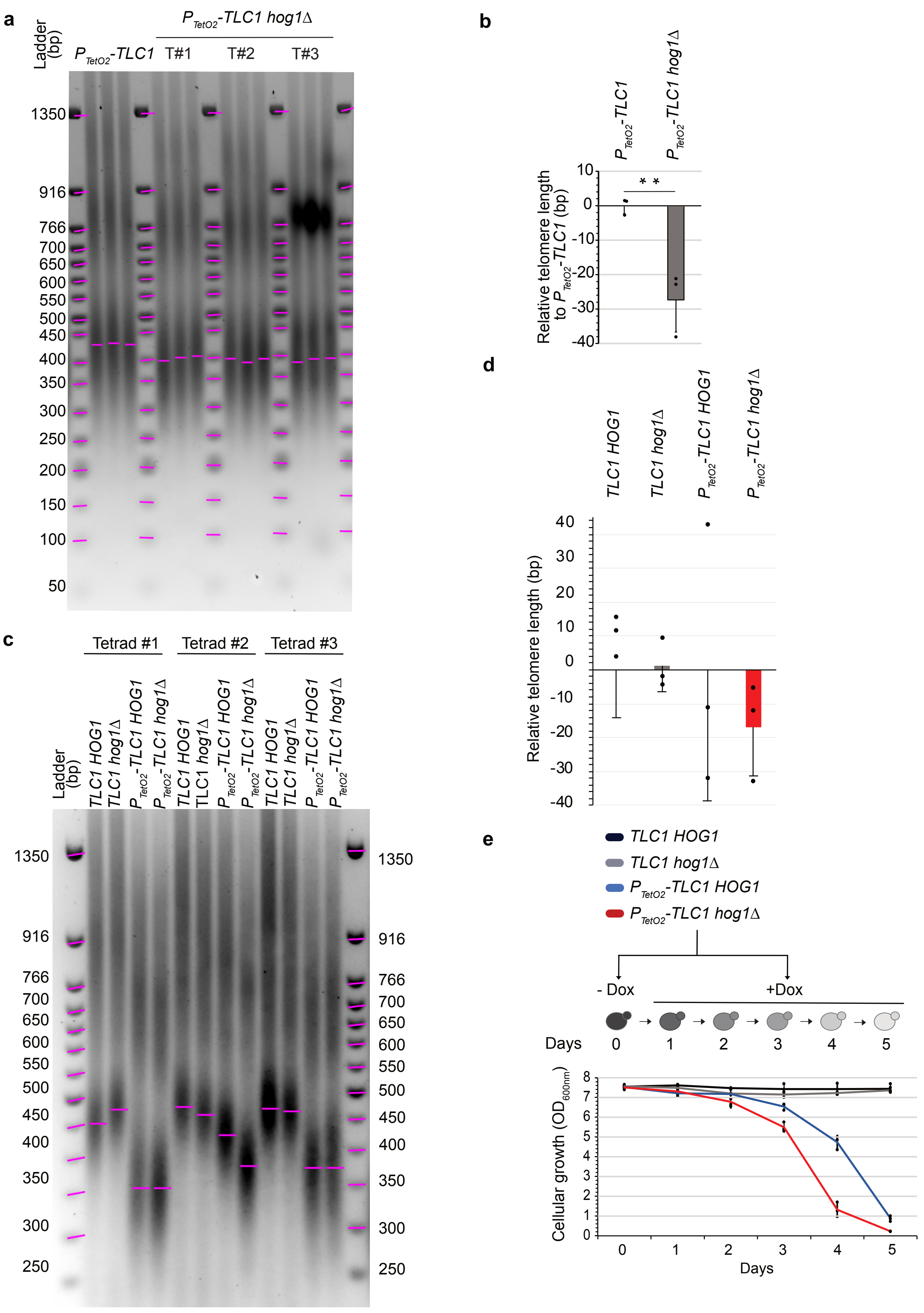

Supplementary Figure 5: No correlation between the acceleration of senescence and the initial shorter size of telomeres in the absence of Hog1. (a) Technical triplicates of telomere-PCR of Y' telomeres from the independent transformants (T#1-3) and strains indicated and **(b)** their quantification, plotted as mean ± SD . P-values were calculated by two-tailed Student's t-test (** < 0,01). **(c)** Telomere-PCR of Y' telomeres from clones with indicated genotypes at D0 originating from three tetrads dissection and **(d)** their quantification normalized to the *TLC1 HOG1* strain (WT) for *TLC1 hog1*Δ (*hog1*Δ*) versus* to the P_{TetO2}-*TLC1 HOG1* for the P_{TetO2}-*TLC1 hog1*Δ, plotted as mean ± SD. **(e)** Cell Density at OD_{600nm}. Cells from the tetrads dissection corresponding to S5c with the genotypes indicated were treated with doxycycline as described in Figure 1.

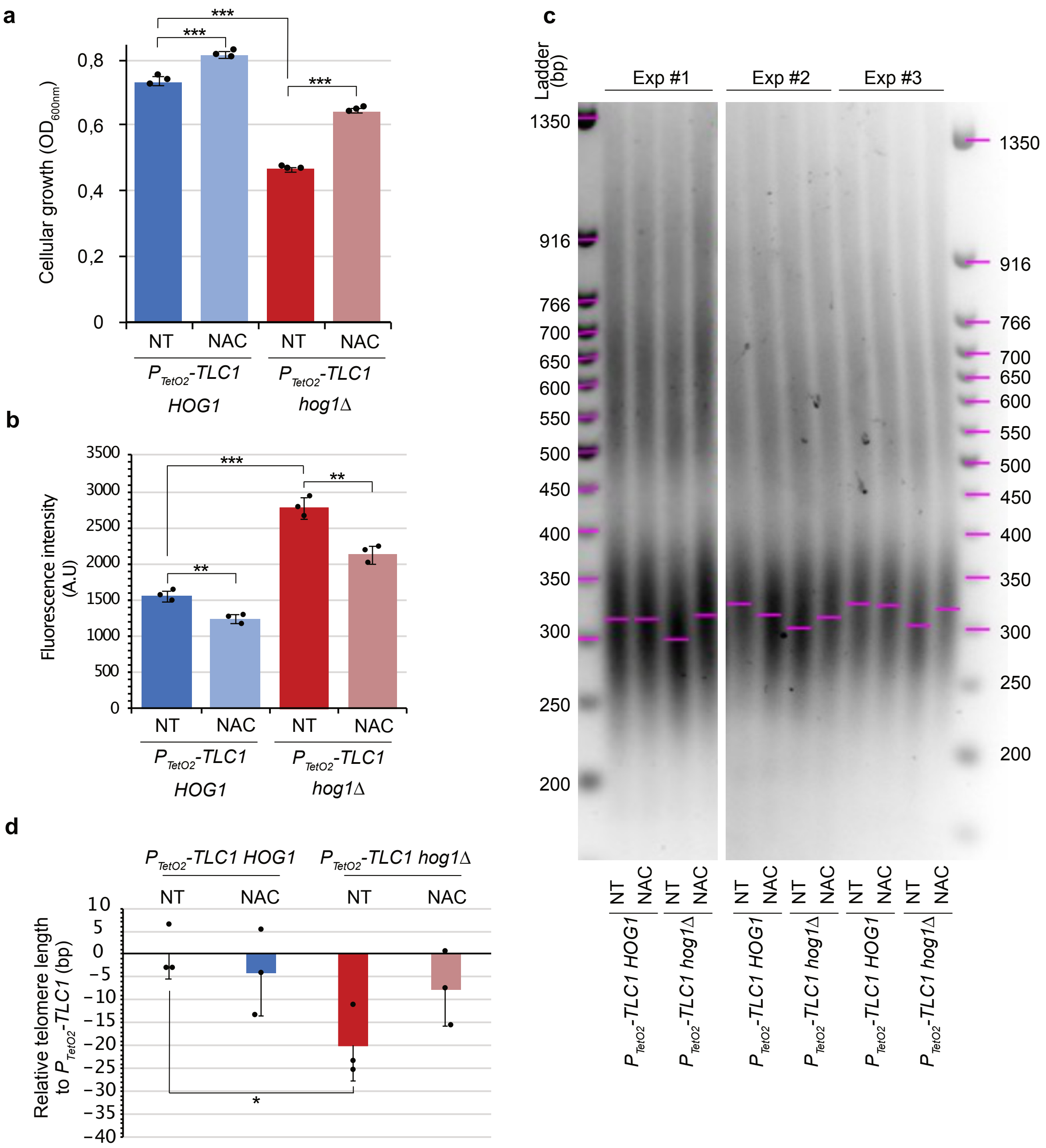

Supplementary Figure 6: The effect of NAC treatment on proliferation and telomeres length.
Cells with the genotype indicated were diluted at 0,00033 of OD and grown in media without or with NAC 10 Mm over 10 generations in exponential phase. **(a)** Cell density at OD_{600nm} and **(b)** ROS level were measured after 10 generations and are plotted as mean ± SD of three independent experiments. **(c)** Experimental triplicates of telomere-PCR of Y' telomeres on samples from experiment 6a and **(d)** their quantification, plotted as mean ± SD. P-values were calculated by two-tailed Student's t-test (* < 0,05; ** < 0,01; ***< 0,001) and only significant differences have been represented.

Material and methods for supplementary figure 7:

Cells harbouring a biosensor Cytorosella and Mitorosella consisting of a rapidly maturing, pH-stable red fluorescent protein fused with a pH-sensitive variant of green fluorescent protein. Its function depends on variations in pH across various cellular compartments and the vacuole. the Cytorosella and Mitorosella constructions are used to follow autophagy and mitophagy respectively. Cells were diluted to 0,1 OD_{600nm} respectively in SD-Leu and SG-Leu at 30 °C under 220 rpm rotation. When cells reached 0,8 OD, they were washed 3 times with water and transferred to SD-N media for 24 hours to induce autophagy or mitophagy then samples for microscopy analysis were taken. Images were captured using a fully motorized Axio Observer Z1 inverted microscope (Zeiss) with DsRed, GFP and Phase settings.

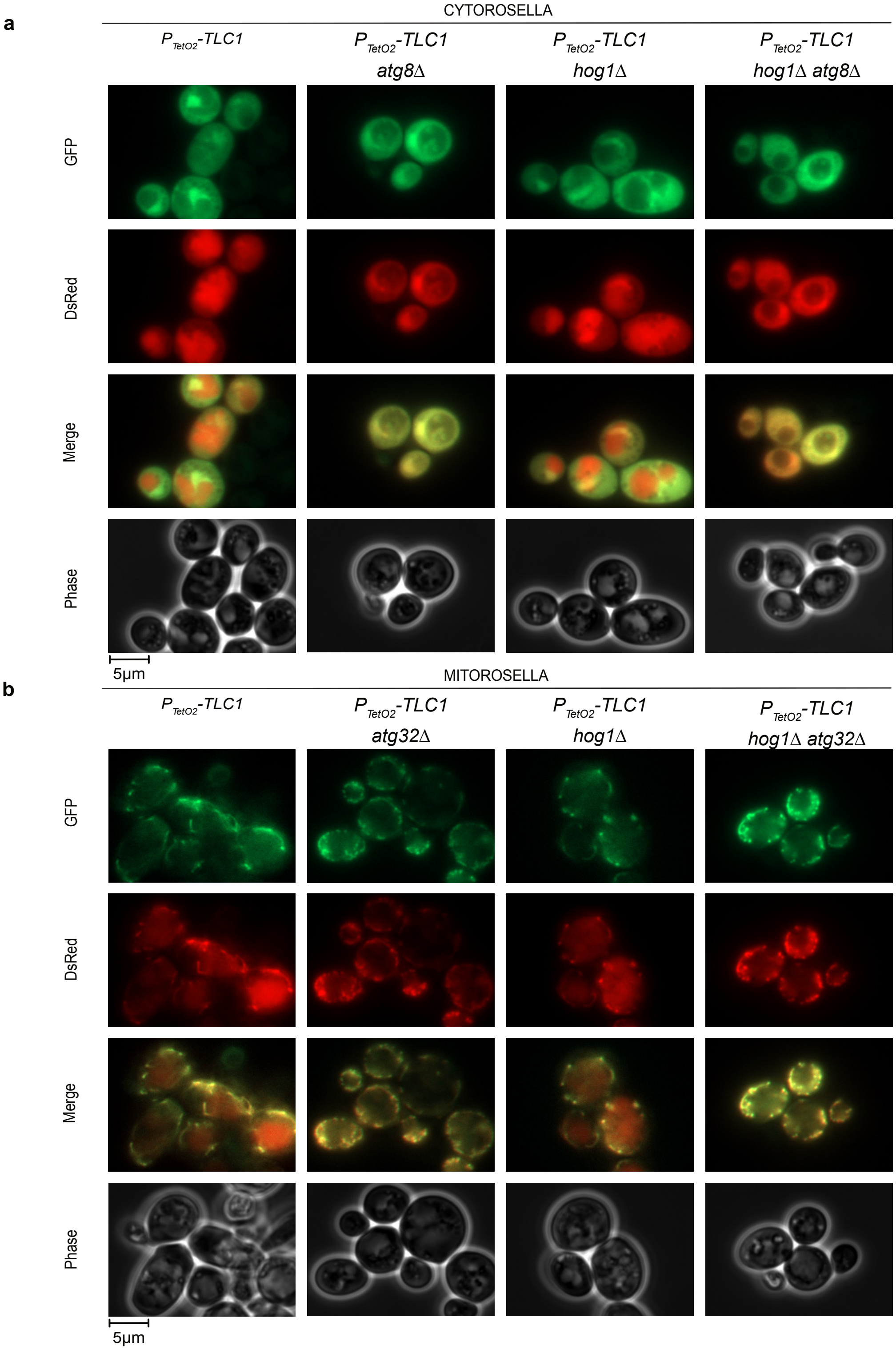

Supplementary Figure 7: Strains deleted for *HOG1* do not affect autophagy and mitophagy phenotype. Cytorosella is a fusion between a cytoplasmic targeting signal, a DsRed, and a GFP sensitive to pH. Mitorosella is similar to cytorosella but with a mitochondrial targeting signal. Images of fluorescence microscopy following autophagy **(a)** and mitophagy **(b)** induction by nitrogen depletion for 24 hours. **(a)** P_{TetO2}-*TLC1* and P_{TetO2}-*TLC1 hog1*Δ strains transformed with Cytorosella exhibit red fluorescence accumulation in the vacuole reflecting autophagy process, and lack green fluorescence, indicating the delivery of Cytorosella to the vacuole. Conversely, P_{TetO2}-*TLC1 atg8*Δ and P_{TetO2}-*TLC1 hog1*Δ *atg8*Δ strains, used as a positive control, do not demonstrate accumulation of either fluorophore in the vacuole. **(b)** For mitophagy process, similarly, following 24 hours of nitrogen depletion, P_{TetO2}-*TLC1* and P_{TetO2}-*TLC1 hog1*Δ strains transformed with Mitorosella display red fluorescence accumulation within the vacuole while lacking green fluorescence, indicating the delivery of mitochondria to the vacuole. Conversely, P_{TetO2}-*TLC1 atg32*Δ and P_{TetO2}-*TLC1 hog1*Δ *atg32*Δ strains, used as a positive control, do not exhibit accumulation of either fluorophore in the vacuole.

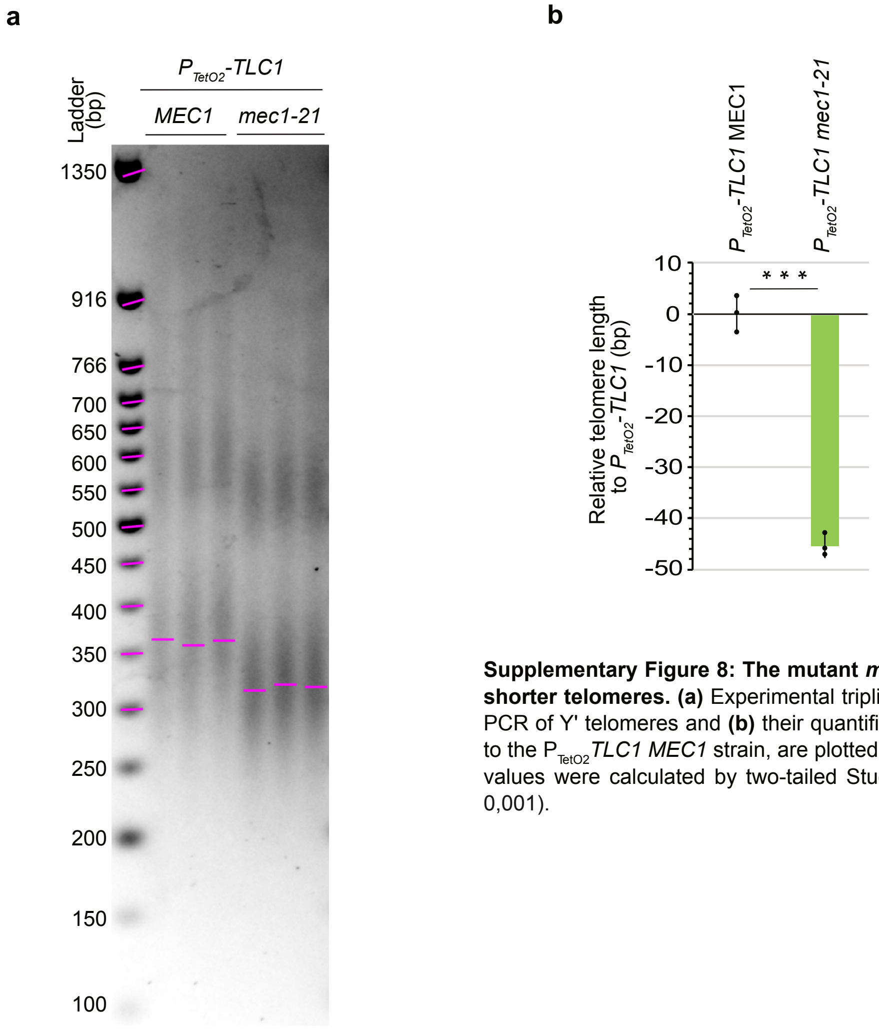

Supplementary Figure 8: The mutant *mec1-21* results in shorter telomeres. (a) Experimental triplicates of telomere-PCR of Y' telomeres and **(b)** their quantification, normalized to the P_{TetO2}*TLC1 MEC1* strain, are plotted as mean ± SD. P-values were calculated by two-tailed Student's t-test (*** < 0,001).

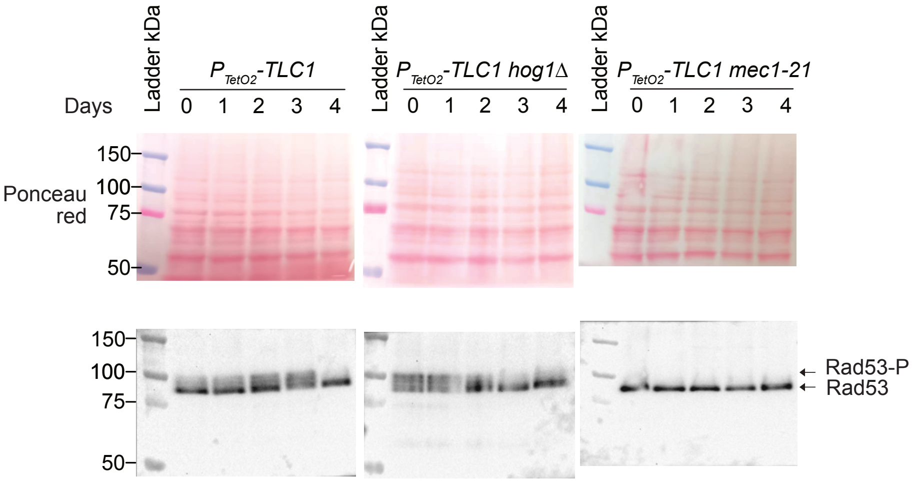

Supplementary Figure 9: Uncropped scans of Western blot relative to Figure 4c. Protein extracts analysed by Western blot using an antibody against Rad53.
